# What Every Cardiologist Should Know about H1N1?

**Published:** 2010

**Authors:** Allahyar Golabchi, Nizal Sarrafzadegan

**Affiliations:** 1 MD; Resident of Cardiology, Department of Cardiology, School of Medicine, Isfahan University of Medical Sciences, Isfahan, Iran; 2 MD; Professor of Cardiology, Isfahan Cardiovascular Research Center, Isfahan University of Medical Sciences, Isfahan, Iran

**Keywords:** H1N1 virus, Vaccination, Cardiovascular disease, Myocarditis

## Abstract

The world is witnessing ever fastest growing pandemic with high morbidity and mortality that excessive volume of airline travels spread influenza infection; so physicians with various specialties should know and consider the impact of current pandemic on their daily practice. Among influenza A viruses that infect humans, an influenza A virus emerged that had shifted to H1N1. Primarily, the results of pandemic of H1N1 were in younger humans without preexisting immunity. Attack rates of swine influenza are relatively high, but mortality is relatively low and mortality rate is highest in the very young, the very old, and the immunosuppressed. In this new pandemic, there is not more evidence of the interface of H1N1 with chronic diseases; however, we expect that the Swine flu such as the previous influenza pandemics can change the course of many chronic diseases.

In this review, we want to show the impacts of swine flu on cardiovascular system and disease. We will also discuss the importance of vaccination in chronic cardiovascular disease.

## Introduction

The world is witnessing ever fastest growing pandemic with high morbidity and mortality that excessive volume of airline travels spread influenza infection^1^; so physicians with various specialties should know and consider the impact of current pandemic on their daily practice. Among influenza A viruses that infect humans, three major subtypes of hemagglutinins (H1, H2, and H3) and two subtypes of neuraminidases (N1 and N2) have been described. In 1977, an influenza A virus emerged that had shifted to H1N1. Primarily, the results of pandemic of H1N1 were in younger humans without preexisting immunity. Attack rates of swine influenza are relatively high, but mortality is relatively low and mortality rate is highest in the very young, the very old, and the immunosuppressed.^2^ Influenza A prevalence begins abruptly, peak over a two to three week period, and last for two to three months, classically.^3^ Most outbreaks have incidence rates of 10 to 20 percent in the general population, but rates can exceed 50 percent in pandemics.^4^ Tsibane et al. showed that people born in or before 1915 had neutralizing antibody responses to the swine influenza strain derived from the B cells that caused presumably, the 1918 pandemic, even ninety years after its outbreaks. By genetically methods, they also cross-reacted with similar hemagglutinins of a 1930 H1N1 influenza.^5^ In 1918 and 1919 pandemic of swine flu resulted in approximately 20 to 50 million deaths worldwide and was exceptionally high death among healthy adults aged 15 to 34 years^6^; also, in 2009 pandemic, near one-third of severe cases had no underlying conditions with lower median age than the previous seasonal flu.^7^ In this new pandemic, there is not more evidence of the interface of H1N1 with chronic diseases; however, we expect that the swine flu such as the previous influenza pandemics can change the course of many chronic diseases.

In this review, we want to show the impact of swine flu on cardiovascular system and disease. We will also discuss the importance of vaccination in chronic cardiovascular disease.

### Myocarditis by flu virus

The majority of our knowledge about influenza myocarditis came from isolated case reports and series. The incidence rate of the influenza A myocarditis was 9% in the study of Karjalainen et al.^8^ Perimyocarditis typically occur between 4 and 9 days after the onset of influenza symptoms with worsening dyspnea. Electrocardiogram may show new changes, such as: ST elevation, Q waves and Left Bundle Branch Block. Cardiac enzyme (CK-MB and Troponin I) levels elevate in all the patients and reduced left ventricular function exists in most patients. Even fulminant myocarditis can occur with a distinct onset always within the first 2 weeks. Some patients present with profound left ventricular dysfunction. The endomyocardial biopsy shows multiple foci of active inflammation and necrosis. Patients recover or die within 2 weeks with complete histological and functional recovery of the myocardium.^9^ Influenza myocarditis can result in the development of a dilated cardiomyopathy as a late squeal.^10^ Pericardial effusion can exist with significant volume to cause cardiac tamponade.^11^ In H1N1infected children has reported high incidence of myocarditis, so early detection and aggressive management are paramount.^12^ Increasing the awareness of influenza myocarditis may help in the earlier detection and treatment of this disease during influenza epidemics.

### Acute coronary syndrome and flu virus

The influenza A infection showed a rise in Acute Myocardial Infarction (AMI), Chronic Ischemic Heart Disease (IHD) and subsequently mortality during epidemics. This effect was observed in both genders at all age groups. A study in the United States have previously estimated that influenza causes up to 92000 deaths per year by triggering AMI.^13^ Generally, cardiologists whose patients have had influenza followed by fatal myocardial infarction reported myocardial infarction mortality related to traditional risk factors not Influenza infection, which is a neglected risk factor.^14^ The first 3–5 days within influenza infection is the highest risk time for AMI.^15^ Also, in patients with influenza and pneumonia, AMI may be missed as findings of dyspnea, chest pain, fever and leucocytosis are considered related to pneumonia alone.

### Mechanism of atherogenesis by flu virus

The positive correlation between antibodies to influenza A virus and antibodies to oxidized low density lipoproteins titers show that activated autoimmune system may lead to the susceptibility to atherosclerosis.^16^ Other proposed potential mechanisms include: (1) an increase in pro-inflammatory, prothrombotic cytokines; (2) endothelial dysfunction; (3) increased plasma viscosity; (4) psychological stress; (5) decreased supply of the heart such as dehydration leading to hypotension; (6) increased demand of the heart such as tachycardia; (7) loss of the anti-inflammatory properties of HDL particles; (8) increase in invasion of macrophages into the arterial wall; (9) reduction in clotting time due to pronounced expression of inflammatory cytokines by infected monocytes; (10) atherosclerotic changes in the walls of arteries in patients with influenza and apolipoprotein-E deficiency.^17^–^20^

### Treatment

We can use the new neuraminidase-inhibitors such as oseltamivir (Tamiflu) and zanamivir (Relenza) in patients with silent or clinical symptoms who are exposed to influenza, but did not receive the influenza vaccine or their immune response to the vaccine is inadequate.^2^ Studies suggest that oseltamivir treatment for influenza is associated with significant decrease in mortality in patients with history of cardiovascular disease.^21^ In influenza myocarditis, treated patients with intravenous ribavirin has demonstrated that influenza viral titres declined abruptly following the initiation of therapy^22^, although case reports have showed that supportive treatment with plasmapheresis has been successful .^23^ In trials on mice, immunoglobulin therapy suppressed influenza A virus myocarditis by increasing neutralizing titers.^24^ We can use inotropic agents and other left ventricular support devices in patients presenting with cardiogenic shock.

### Flu vaccine in organic heart disease

In influenza pandemics, cardiovascular death surpassed other causes of mortality.^25^ Studies show an association between influenza vaccination with reduced risk of non-fatal MI^26^, recurrent ischemic events in patients suffering from infarction or post-angioplasty during flu season.^27^ Naghavi et al. cleared that influenza vaccination was associated with a 67% reduction in the risk of MI in the subsequent influenza seasons.^28^ In a study, influenza vaccination of schoolchildren resulted in a decline in the incidence of influenza and attributed mortality among older people.^29^ During influenza seasons, Nichol et al. showed that vaccination against influenza in people aged more than 65y, reduced hospitalization for heart diseases and cerebrovascular diseases by 19% and 23% respectively. Also, all-cause mortality rate was reduced by 50%.^30^ The American Heart Association (AHA) and the American College of Cardiology (ACC) recommended influenza vaccination in children and adults with coronary and other atherosclerotic vascular diseases as part of secondary prevention^31^, also no harmful effects were seen in individuals with chronic cardiovascular diseases who received influenza vaccination.^32^ Vaccination in patients on long-term anticoagulant therapy was reported to be safe too.^33^ Inactivated influenza vaccines can be administered safely to heart transplant recipients without an increased incidence of rejection or infection.^34^, ^35^ Vaccination is also administrated for individuals who might transmit influenza to at high risk people, such as healthcare workers, providers of home care and household contacts of people in high risk groups.^36^ Additional protection during an influenza outbreak should be the use of plaque-stabilizing agents such as statins, beta-blockers, aspirin, and angiotensin-converting enzyme inhibitors.^26^

## Conclusion

In summary, there is mounting evidence that influenza especially in epidemics and pandemics can trigger myocardial infarction, stroke, and sudden cardiac death. The role of influenza in cardiovascular disease is neglected in Cardiology textbooks and practice of the physicians. Furthermore, influenza vaccine is cost-effective for some groups such as chronic cardiovascular diseases. Rates of vaccination are below the optimal level in most countries. To increase influenza vaccination of patients at high risk groups an intense public health effort is needed and cardiologists need to improve vaccination rates by actively advocating vaccination following the recent AHA/ACC guidelines. Also, special attention should be paid to symptoms and signs of high risk cardiovascular patients who have an upper respiratory tract infection because direct myocardial involvement is not rare in any pandemics of influenza. We conclude that broadened indications for influenza vaccination and treatment, together with targeted prevention efforts, will save many people with cardiovascular diseases.
